# Modeling outcome trajectories in patients with acquired brain injury using a non-linear dynamic evolution approach

**DOI:** 10.1038/s41598-023-33560-x

**Published:** 2023-04-18

**Authors:** Simona Panunzi, Lucia Francesca Lucca, Antonio De Tanti, Francesca Cava, Annamaria Romoli, Rita Formisano, Federico Scarponi, Anna Estraneo, Diana Frattini, Paolo Tonin, Ilaria Piergentilli, Giovanni Pioggia, Andrea De Gaetano, Antonio Cerasa

**Affiliations:** 1 CNR-IASI, Laboratorio di Biomatematica, Consiglio Nazionale delle Ricerche, Istituto di Analisi dei Sistemi ed Informatica, Rome, Italy; 2 S. Anna Institute, Crotone, Italy; 3 Cardinal Ferrari Rehabilitation Centre, Fontanellato, PR Italy; 4 Rehabilitation Institute Montecatone, Montecatone, Imola, BO Italy; 5 IRCCS- Don Carlo Gnocchi Foundation, Florence, Italy; 6 Neurorehabilitation 2 Unit, IRCCS Fondazione Santa Lucia, Rome, Italy; 7 Department of Rehabilitation, San Giovanni Battista Hospital, Foligno, PG Italy; 8 Department of Rehabilitation, Vimercate Hospital, Vimercate, MB Italy; 9 IRIB-CNR, Institute for Biomedical Research and Innovation, National Research Council, 98164 Messina, Italy; 10 Department of Biomatics, Óbuda University, Budapest, Hungary; 11 Pharmacotechnology Documentation and Transfer Unit, Preclinical and Translational Pharmacology, Department of Pharmacy, Health Science and Nutrition, University of Calabria, 87036 Arcavacata, CS Italy

**Keywords:** Data processing, Predictive medicine, Neurology, Mathematics and computing

## Abstract

This study describes a dynamic non-linear mathematical approach for modeling the course of disease in acquired brain injury (ABI) patients. Data from a multicentric study were used to evaluate the reliability of the Michaelis–Menten (MM) model applied to well-known clinical variables that assess the outcome of ABI patients. The sample consisted of 156 ABI patients admitted to eight neurorehabilitation subacute units and evaluated at baseline (T0), 4 months after the event (T1) and at discharge (T2). The MM model was used to characterize the trend of the first Principal Component Analysis (PCA) dimension (represented by the variables: feeding modality, RLAS, ERBI-A, Tracheostomy, CRS-r and ERBI-B) in order to predict the most plausible outcome, in terms of positive or negative Glasgow outcome score (GOS) at discharge. Exploring the evolution of the PCA dimension 1 over time, after day 86 the MM model better differentiated between the time course for individuals with a positive and negative GOS (accuracy: 85%; sensitivity: 90.6%; specificity: 62.5%). The non-linear dynamic mathematical model can be used to provide more comprehensive trajectories of the clinical evolution of ABI patients during the rehabilitation period. Our model can be used to address patients for interventions designed for a specific outcome trajectory.

## Introduction

Brain injury is the leading cause of death and disability worldwide. The degree of severity varies according to a combination of several demographics (i.e., age), etiology (vascular, traumatic or anoxic), clinical (i.e., time since injury, degree of disability at admission), radiological (i.e., side and size of the lesion), cognitive, behavioral, psychosocial, and environmental issues, which can interfere with the effectiveness of rehabilitation interventions and, thus, with the final outcome at discharge^1^. The Global Burden of Diseases, Injuries, and Risk Factors Study (GBD) reported that in the last 26 years the global incidence has increased by 8.4% in patients with Traumatic Brain Injury (TBI) (Traumatic Brain Injury and Spinal Cord Injury Collaborators^2^, but has decreased by 8.1% in stroke patients (Stroke Collaborators^3^).

Identifying reliable tools for the early prediction of outcomes is the main target for physicians in guiding care decisions in patients with ABI^4,5^. In the last 10 years, the application of machine learning (ML) algorithms has increased rapidly in this field^6^, but with a poor translation to clinical practice. This is basically due to the uncertainty regarding the intrinsic ‘‘black-box’’ nature of this approach and the lack of clear advantages with respect to traditional approaches (i.e. linear regression models)^7^. In fact, a recent narrative review showed that ML algorithms do not outperform traditional regression approaches for outcome prediction in brain injury^8^.

The current trend to shorten the length of stay in rehabilitation units, as well as the increasing number of new cases and demand for efficiency in care, suggest that knowledge of the prognosis for the outcome is crucial to optimize the management of ABI patients in the first months after brain injury^9^. A new statistical approach that better describes the dynamics of clinical changes occurring during the entire neurorehabilitation period is therefore the greatest unmet need for clinical practice. A new computational approach should provide individual predictions of the clinical course and depend on solid predictors coupled with each clinical intervention involved in neurorehabilitation. A dynamic model that accounts for the nonlinearity of the clinical evolution characterized by a high variability in clinical signs, symptoms, and treatments would be the preferred approach.

The advent of mathematical models used to forecast the clinical course in severe neurological patients could play a new pivotal role, for several reasons. First, to condense the multidimensional complexity of neurodegenerative mechanisms into model elements designed to capture the key physiological features, with a clear correspondence between the mathematical representation of the clinical evolution, and the effect of medical therapy. In addition, in order to provide a clear quantitative depiction of pathophysiological pathways, models have been developed to indirectly calculate physiologically or clinically relevant parameters from experimental data. A first attempt to apply mathematical modeling to predict clinical course of acquired brain injury was made by Srinivasan et al.^10^. They created an index combining the predictive values of the Glasgow coma scale (GCS) with the presence of Fronto-Orbicular Reflex and Vertical Oculocephalic Response. This preliminary model was prospectively validated on a small sample and reached an accuracy of 90% when used on the third post-traumatic day. Tilling et al.^11^ used a multilevel growth curve model to estimate the effect on the functional recovery of patient characteristics and stroke severity variables at baseline and how these effects varied over time. The typical recovery curve for patients with a given set of attributes is predicted by a multilayer growth curve model. It is assumed that each patient's genuine recovery curve is unique to them and that all patients deviate somewhat from the average recovery curve. Because of this, even among individuals with the same starting characteristics, the models allow for different recovery paths. Validating prospective data on 299 stroke patients, these authors demonstrated that age, dysphasia, and limb deficiency also had an impact on the rate of recovery. Urinary incontinence, sex, prestroke impairment, and dysarthria all had an impact on the level of result after stroke. When patients' recovery histories were taken into account, the predicted Barthel Index improved for 69% of the time, lying within 3 points of the observed Barthel index on 49% of the instances. Lastly, using ordinary differential equations, Vaughan et al.^12^ reported interactions between pro- and anti-inflammatory cytokines, M1- and M2-like microglia, and central nervous system (CNS) tissue damage in patients with TBI. This new approach enabled the authors to develop a framework for investigating relationships between acute neuroinflammatory components and patient prognosis in severe TBI by constructing a mathematical characterization of inflammatory processes informed by clinical data.

On this basis, this multicenter study is aimed to develop a new dynamic model applied to ABI patients characterized by a high variability in clinical signs, symptoms, and treatments during the inpatient intensive rehabilitation care period. The proposed approach combines a principal component analysis (PCA) for dimension reduction (capturing the maximum amount of information and reducing the dimensionality problem) and a nonlinear mathematical modeling for describing the evolution of the clinical course in terms of the resulting new PCA dimensions. The nonlinear mathematical formulation employed is a Michaelis–Menten (MM) equation. The MM model is widely used in enzyme kinetics to describe the rate of enzymatic reactions, which was first proposed by Michaelis and Menten^13^. The model describes the relationship between the initial reaction rate (v) and the substrate concentration ([S]) for an enzyme-catalyzed reaction. The model assumes that the reaction follows a simple two-step process, where the substrate first binds to the enzyme to form an enzyme–substrate complex, which then converts into the product and releases the enzyme. This process is represented by the following equation:

$${\text{E}} + {\text{S}} \rightleftharpoons {\text{ES}} \to {\text{E}} + {\text{P}}$$

where E represents the enzyme, S represents the substrate, ES represents the enzyme–substrate complex, and P represents the product. The MM model assumes that the reaction rate is limited by the formation of the enzyme–substrate complex, and that the conversion of the complex into the product is very rapid compared to the formation of the complex. This enables the reaction rate to be described by a simple equation:

$${\text{v}} = \left( {{\text{Vmax}}\left[ {\text{S}} \right]} \right)/\left( {{\text{Km}} + \left[ {\text{S}} \right]} \right)$$

where Vmax is the maximum rate of the reaction, Km is the Michaelis constant, which represents the substrate concentration at which the reaction rate is half of the maximum rate, and [S] is the substrate concentration. The MM equation is widely used to analyze enzyme/pharmaco kinetics data and to estimate the values of Vmax and Km^14^.

However, the MM mathematical model has been applied in other scientific domains as well as enzyme kinetics. For example, it has been used in pharmacokinetics to describe the relationship between drug concentration and its elimination rate from the body. In this case, the model assumes that the drug is eliminated by a first-order process, and the rate of elimination is proportional to the drug concentration^15^. The Michaelis–Menten equation can be adapted to describe this process by replacing [S] with the drug concentration and Km with the concentration at which the elimination rate is half of the maximum rate. Again, the MM equation has also been used in ecology to describe the relationship between resource uptake and resource concentration in organisms^16^. The model assumes that organisms have a maximum uptake rate for a resource, and that the uptake rate decreases as the resource concentration increases due to resource saturation. This can be described by a modified Michaelis–Menten equation which replaces Vmax with the maximum uptake rate and Km with the resource concentration at which the uptake rate is half of the maximum rate. Overall, the Michaelis–Menten model has been used in various fields, where the relationship between an independent variable and the process of interest is described by a saturation curve.

Here, we applied for the first time a slightly modified version of this mathematical model in the present neurological domain for describing the trajectories (evolution) over time (the independent variable) of a predictor, expressing the clinical status of an ABI patient, and characterized by the values or the presence/absence of typical features. The mathematical description of the evolution of ABI patient clinical status allows the prediction of the final outcome. In this mathematical formalization, the time at which the clinical evaluations are performed assumes the role of the substrate concentration in the classical MM equation, the reaction velocity is represented by a predictor summarizing the patient clinical status, Vmax is the maximum increment in the clinical condition attainable before the discharge from the IRU and Km is the required time at which the half of the maximum increment is achieved.

## Results

### Clinical data

As shown in our previous work (Table 1,^17^), 156 ABI patients (99 males, mean age: 53.7 years [range 22–89]) were enrolled in this study. The mean length of stay in the intensive care unit (ICU) was 48.1 days (range 10–144), whereas in the intensive rehabilitation unit (IRU), ABI patients were discharged after an average period of 167.1 days (range 46–600). In the entire cohort, most patients were characterized by vascular pathology (no. 79; 50.6%), followed by traumatic (no. 56; 35.9%), anoxic (no. 15; 9.6%) or other pathologies (i.e. infections/tumour; no. 6; 3.8%) Thirteen patients were discharged before the end of the study (T1 phase). Thus, the final sample for statistical analysis this consisted of 143 patients.

**Table 1:** Means and standard deviations of continuous demographic and clinical predictors at baseline (T0), after 4 months from the event (T1) and at discharge (T2) according to the GOS binary categorization (positive/negative).

Variable	Time	Negative GOS	Positive GOS	*P* value
		Mean ± SD	Mean ± SD	
Age	T0	55.8 ± 17.5	41.7 ± 15.9	< 0.001
ICU days	T0	47.8 ± 20.4	34.6 ± 18.1	0.008
CRS-R	T0	10.74 ± 6.72	17.48 ± 5.9	< 0.001
	T1	14.79 ± 7.14	21.78 ± 2.86	< 0.001
	T2	16.38 ± 7.03	22.71 ± 0.81	< 0.001
RLAS	T0	2.92 ± 1.16	4.26 ± 1.32	< 0.001
	T1	3.77 ± 1.45	6.26 ± 1.1	< 0.001
	T2	4.27 ± 1.27	6.85 ± 1.49	< 0.001
ERBI-A	T0	− 178.63 ± 32.52	− 175 ± 68.3	0.79
	T1	− 146.96 ± 69.45	− 43.42 ± 55.2	< 0.001
	T2	− 109.15 ± 70.23	-9.26 ± 23.15	< 0.001
ERBI-B	T0	0.17 ± 1.13	2.59 ± 12.5	0.32
	T1	2.61 ± 20.68	61.05 ± 33.2	< 0.001
	T2	9.02 ± 17.63	86.67 ± 20.8	< 0.001
PSH-AM-CFS	T0	3.47 ± 2.98	2.48 ± 1.93	0.04
	T1	2.23 ± 2.62	1.26 ± 1.7	0.05
	T2	1.36 ± 2.12	0.52 ± 1.01	< 0.001
PSH-AM-DLT	T0	2.74 ± 2.46	2.56 ± 2.24	0.71
	T1	2.2 ± 2.17	1.58 ± 0.96	0.05
	T2	1.65 ± 1.54	1.15 ± 0.46	< 0.001
PSH-AM	T0	6.2 ± 5.18	4.96 ± 4.05	0.18
	T1	4.42 ± 4.5	2.84 ± 2.3	0.03
	T2	3.02 ± 3.49	1.67 ± 1.33	< 0.001

*GOS* Glasgow outcome scale, *ICU* intensive care unit, *CRS-r* coma recovery scale-revised, *RLAS* Rancho Los Amigos Scale, *ERBI* early rehabilitation Barthel index, *PSH-AM* paroxysmal sympathetic hyperactivity-assessment measure, *CFS* clinical feature scale, *DLT* diagnosis likelihood tool.

At discharge, 2.8% patients had died, 61.1% had a full recovery of consciousness and 36.1% remained in VS/UWS or MCS. The median GOS value was 3 ranging from 1 to 5. At admission CRS-r scores (T0 median value: 9 [1–23]; at discharge: 21 [3–23]) and RLAS scores (T0 median value: 3 [1–27]; at discharge: 5 [1–8]) increased. On the other hand, ERBI values decreased (T0 median value: − 175 [− 50/− 325]; at discharge: − 105 [0/− 175]) compared to admission. PSH signs (including possible/probable) showed an evident decrease during the IRU period (see supplementary materials Table 1).

### Patient characteristics according to the GOS outcome

Tables 1 and 2 report the baseline information and follow-up evaluations according to the GOS binary categorization (positive/negative). One hundred and sixteen (81.1%) out of 143 patients were classified with negative outcome at discharge. Age and ICU days differed significantly between the two groups (*P* < 0.001 and *P* = 0.008; respectively). Patients with a negative GOS were older and showed a higher number of days spent in the ICU compared to patients with a positive outcome. The distribution of males and females in the two GOS groups were similar, 35.3% of patients with a negative outcome were females compared to 44.4% of patients with a positive outcome (*P* = 0.51). Clinical features and use of devices that were found to be significantly different at baseline were as follows: CRS-r (*P* < 0.001), RLAS (*P* < 0.001), PSH-AM-CFS (*P* = 0.04), type of diagnosis (*P* < 0.001), tracheostomy (*P* < 0.001), respiratory function (*P* = 0.03), feeding modality (*P* < 0.001) and fractures (*P* = 0.01). Patients with a negative GOS presented worse clinical conditions in relationship to the above characteristics.

**Table 2:** Absolute frequencies and percentages of categorical demographic and clinical predictors at baseline (T0), after 4 months from the event (T1) and at discharge (T2) according to the GOS binary categorization (positive/negative).

Variables	Groups	Negative GOS		Positive GOS		*P* value
		N	%	N	%	
Sex						
T0	Overall	116	81.12	27	18.88	0.51
	F	41	77.36	12	22.64	
	M	75	83.33	15	16.67	
Etiology						
T0	Overall	116	81.12	27	18.88	0.06
	Anoxic	13	92.86	1	7.14	
	Infectious	2	100	0	0	
	Benign neoplasm	4	100	0	0	
	TBI	35	68.63	16	31.37	
	Vascular	62	86.11	10	13.89	
PSH crisis in intensive care unit						
T0	Overall	116	81.12	27	18.88	0.24
	Presence	10	66.67	5	33.33	
	Absence	106	82.81	22	17.19	
Diagnosis						
T0	Overall	116	81.12	27	18.88	< 0.001
	Emerged from MCS	23	60.53	15	39.47	
	MCS	43	82.69	9	17.31	
	VS/UWS	50	94.34	3	5.66	
T1	Overall	116	81.12	27	18.88	< 0.001
	Death	2	100	0	0	
	Emerged from MCS	51	73.91	18	26.09	
	MCS	29	96.67	1	3.33	
	VS/UWS	26	100	0	0	
	Missing	8	50	8	50	
T2	Overall	116	81.12	27	18.88	< 0.001
	Death	4	100	0	0	
	Emerged from MCS	66	70.97	27	29.04	
	MCS	30	100	0	0	
	VS/UWS	16	100	0	0	
Tracheostomy						
T0	Overall	116	81.12	27	18.88	< 0.001
	Presence	109	85.83	18	14.17	
	Absence	7	43.75	9	56.25	
T1	Overall	116	81.12	27	18.88	< 0.001
	Presence	68	97.14	2	2.86	
	Absence	38	69.09	17	30.91	
	Missing	10	55.56	8	44.44	
T2	Overall	116	81.12	27	18.88	< 0.001
	Presence	48	100	0	0	
	Absence	64	70.33	27	29.67	
	Missing	5	100	0	0	
Respiratory function						
T0	Overall	116	81.12	27	18.88	0.03
	Assisted	4	80	1	20	
	Autonomous	41	70.69	17	29.31	
	Autonomous + o2	71	88.75	9	11.25	
T1	Overall	116	81.12	27	18.88	0.01
	Autonomous	80	80.81	19	19.19	
	Autonomous + o2	25	100	0	0	
	Missing	11	57.89	8	42.11	
T2	Overall	116	81.12	27	18.88	0.01
	Assisted	1	100	0	0	
	Autonomous	90	76.92	27	23.07	
	Autonomous + o2	21	100	0	0	
	Missing	4	100	0	0	
Feeding modality						
T0	Overall	116	81.12	27	18.88	< 0.001
	Oral	3	30	7	70	
	Parenteral	1	33.33	2	66.67	
	PEG	64	90.14	7	9.86	
	NG tube	48	81.36	11	18.64	
T1	Overall	116	81.12	27	18.88	< 0.001
	Oral	33	66	17	34	
	Parenteral	0	0	0	0	
	PEG	63	96.92	2	3.08	
	NG tube	10	100	0	0	
	Missing	10	55.56	8	44.44	
T2	Overall	116	81.12	27	18.88	< 0.001
	Oral	46	63.89	26	36.11	
	Parenteral	0	0	1	100	
	PEG	63	100	0	0	
	NG tube	3	100	0	0	
	Missing	4	100	0	0	
Urinary catheter						
T0	Overall	116	81.12	27	18.88	1
	Presence	113	81.29	26	18.71	
	Absence	3	75	1	25	
T1	Overall	116	81.12	27	18.88	< 0.001
	Presence	55	98.21	1	1.79	
	Absence	51	73.91	18	26.09	
	Missing	10	55.55	8	44.44	
T2	Overall	116	81.12	27	18.88	< 0.001
	Presence	44	100	0	0	
	Absence	68	71.58	27	28.42	
	Missing	4	100	0	0	
Pressure sores						
T0	Overall	116	81.12	27	18.88	0.16
	Presence	45	88.23	6	11.76	
	Absence	71	77.17	21	22.83	
T1	Overall	116	81.12	27	18.88	0.05
	Presence	31	96.88	1	3.12	
	Absence	75	80.65	18	19.35	
	Missing	10	55.55	8	44.44	
T2	Overall	116	81.12	27	18.88	0.05
	Presence	16	94.12	1	5.88	
	Absence	96	78.69	26	21.31	
	Missing	4	100	0	0	
Fractures						
T0	Overall	116	81.12	27	18.88	0.01
	Presence	22	64.71	12	35.29	
	Absence	94	86.24	15	13.76	
T1	Overall	116	81.12	27	18.88	0.9
	Presence	7	77.78	2	22.22	
	Absence	99	85.34	17	14.66	
	Missing	10	55.55	8	44.44	
Severe spasticity						
T0	Overall	116	81.12	27	18.88	0.1
	Presence	22	95.65	1	4.35	
	Absence	94	78.33	26	21.67	
T1	Overall	116	81.12	27	18.88	0.09
	Presence	35	94.59	2	5.41	
	Absence	71	80.68	17	19.32	
	Missing	10	55.55	8	44.44	
T2	Overall	116	81.12	27	18.88	0.09
	Presence	35	97.22	1	2.78	
	Absence	77	74.76	26	25.24	
	Missing	4	100	0	0	
Heterotopic ossification						
T0	Overall	116	81.12	27	18.88	1
	Presence	3	75	1	25	
	Absence	113	81.29	26	18.71	
T1	Overall	116	81.12	27	18.88	0.88
	Presence	12	80	3	20	
	Absence	93	85.32	16	14.68	
	Missing	11	57.89	8	42.11	
T2	Overall	116	81.12	27	18.88	0.88
	Presence	7	70	3	30	
	Absence	105	81.4	24	18.6	
	Missing	4	100	0	0	
Craniectomy						
T0	Overall	116	81.12	27	18.88	0.81
	Presence	25	78.13	7	21.87	
	Absence	91	81.98	20	18.02	
T1	Overall	116	81.12	27	18.88	0.52
	Presence	18	78.26	5	21.74	
	Absence	88	86.27	14	13.73	
	Missing	10	55.55	8	44.44	
T2	Overall	116	81.12	27	18.88	0.52
	Presence	12	100	0	0	
	Absence	100	78.74	27	21.26	
	Missing	4	100	0	0	
Normal-pressure hydrocephalus						
T0	Overall	116	81.12	27	18.88	0.41
	Presence	13	92.86	1	7.14	
	Absence	103	79.84	26	20.16	
T1	Overall	116	81.12	27	18.88	0.37
	Presence	24	92.31	2	7.69	
	Absence	82	82.83	17	17.17	
	Missing	10	55.55	8	44.44	
T2	Overall	116	81.12	27	18.88	0.37
	Presence	26	96.3	1	3.7	
	Absence	86	76.79	26	23.21	
	Missing	4	100	0	0	

*TBI* traumatic brain injury, *MCS* minimally conscious state, *VS/UWS* vegetative state/unresponsive wakefulness syndrome, *PEG* percutaneous endoscopic gastrostomy, *NG tube* nasogastric tube, *PSH* paroxysmal sympathetic hyperactivity.

### Principal component analysis

“Severe spasticity”, “pressure sores”, “POA”, “craniectomy” and “normal-pressure hydrocephalus” variables were not considered in the PCA as they were not found to be significant in a univariable analysis assessing possible differences between GOS groups (positive/negative).

**Figure 1:**
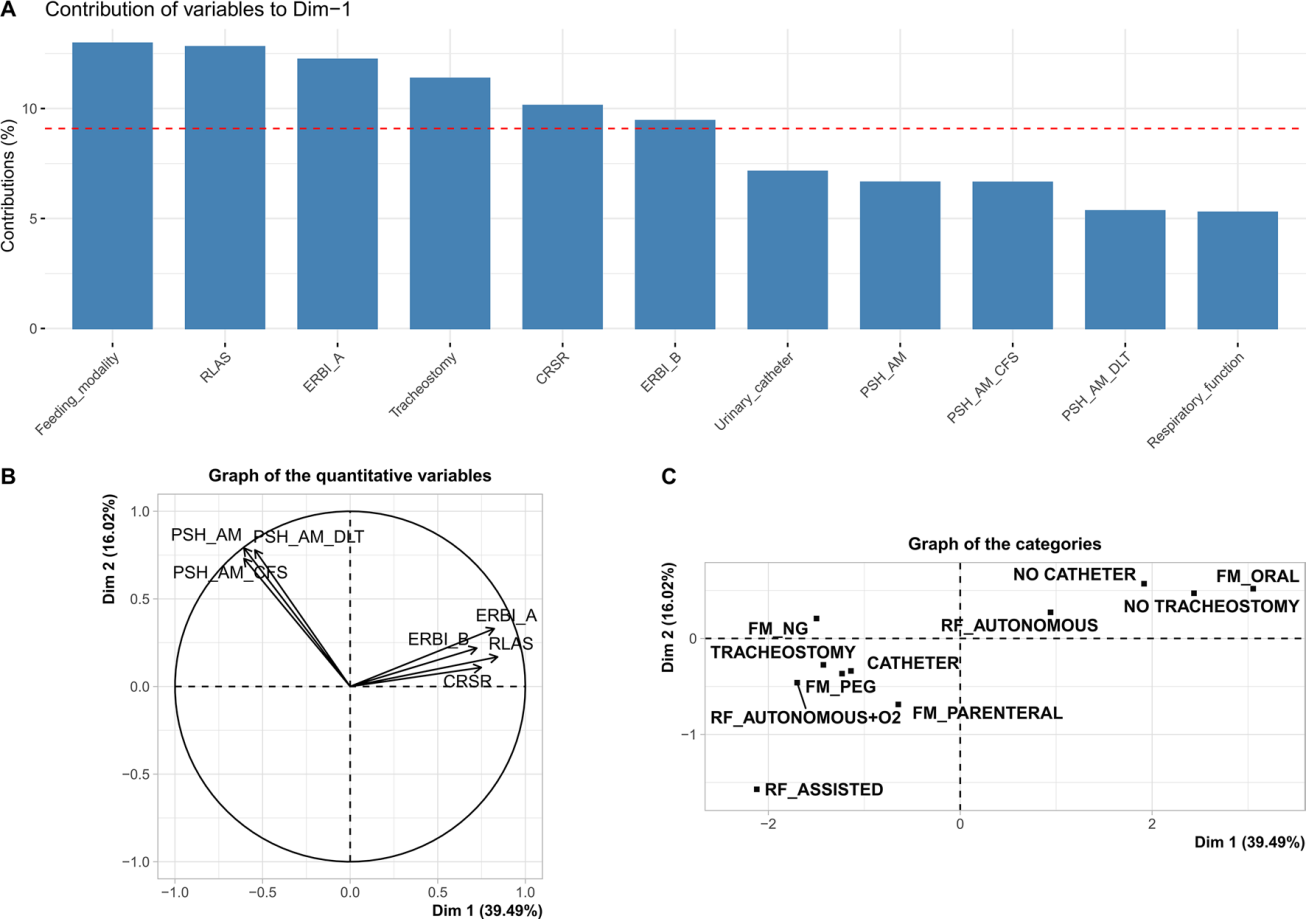
Panel (**A**) Contributions, in percentage terms, of each predictor to the first dimension from the principal component analysis. Panel (**B**) Plot of the loading vectors (coefficients of the variables on the principal components) for quantitative predictors. Panel (**C**) Plot of the coefficients of the variables on the principal components for qualitative predictors.

The first two components from the PCA, performed with the FAMD method, explained 55.5% of the total variance (first component, Dimension 1: 39.5%; second component, Dimension 2: 16.0%). Dimension 1, which explained most of the data variability, was considered in the subsequent analyses. Figure 1 shows the contribution of each variable to the Dimension 1 (Panel A). Dimension 1 was mainly determined by the variables Feeding modality, RLAS, ERBI-A, presence of Tracheostomy, CRS-r, and ERBI-B. Panels B and C show the coefficients of the quantitative and qualitative variables, respectively, on the two dimensions. Dimension 2 was excluded by the modeling approach, as its trend over time did not differentiate between patients with positive and negative outcome.

**Figure 2:**
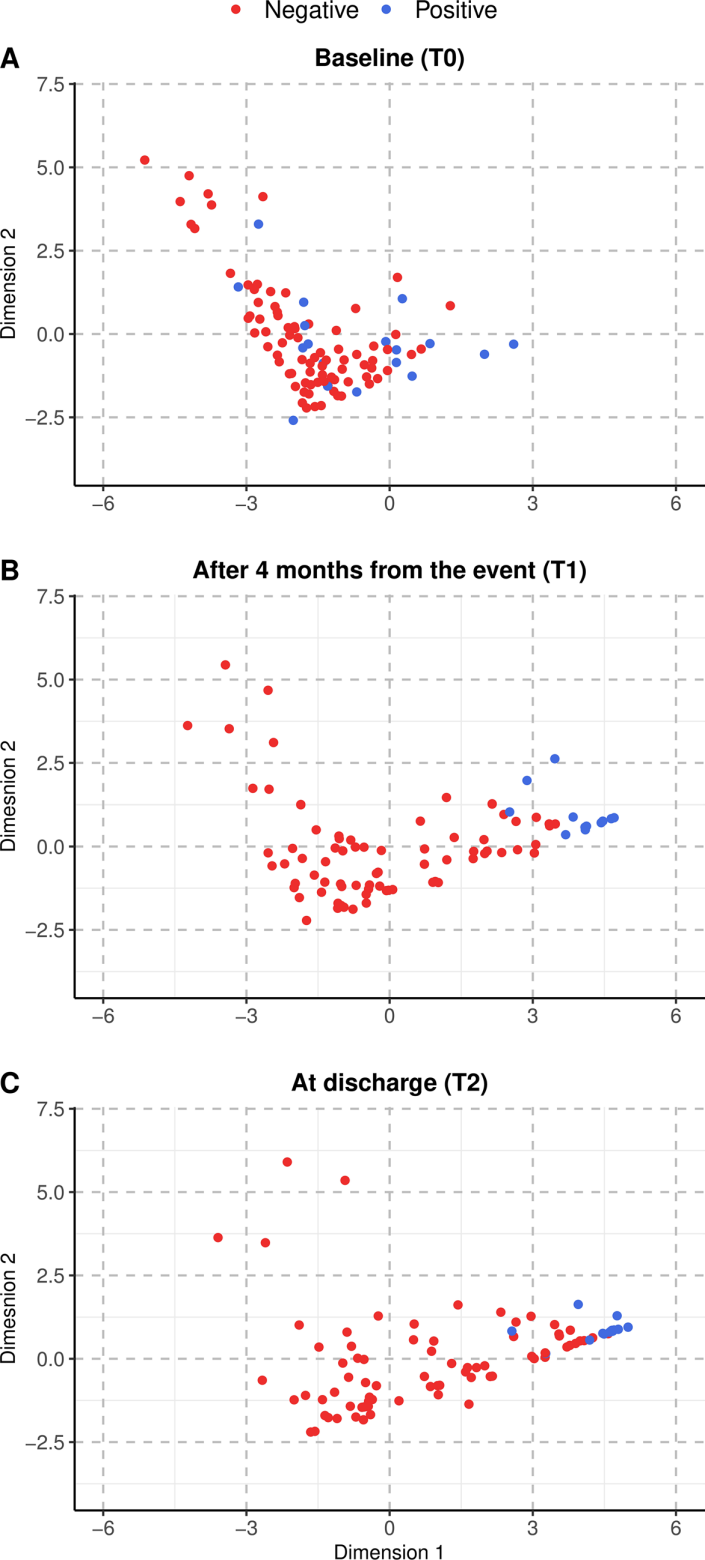
Patients’ scores on the two PCA dimensions at baseline (T0), panel (**A**); 4 months after the event (T1), panel (**B**); at discharge (T2), panel (**C**), for individuals with Positive (blue dots) and Negative (red dots) outcomes.

Figure 2 shows the patient scores regarding each dimension at the three evaluation time points (T0: baseline, T1: 4 months after the event, T2: at discharge). Patients were identified according to their outcome (positive: blue dots, negative: red dots). The figure shows a general increase in the scores over time for Dimension 1, with a larger increase observed for patients with a positive outcome. Patients in the two groups show overlapping scores for Dimension 2.

### Parameter estimation of Michaelis Menten models

**Figure 3:**
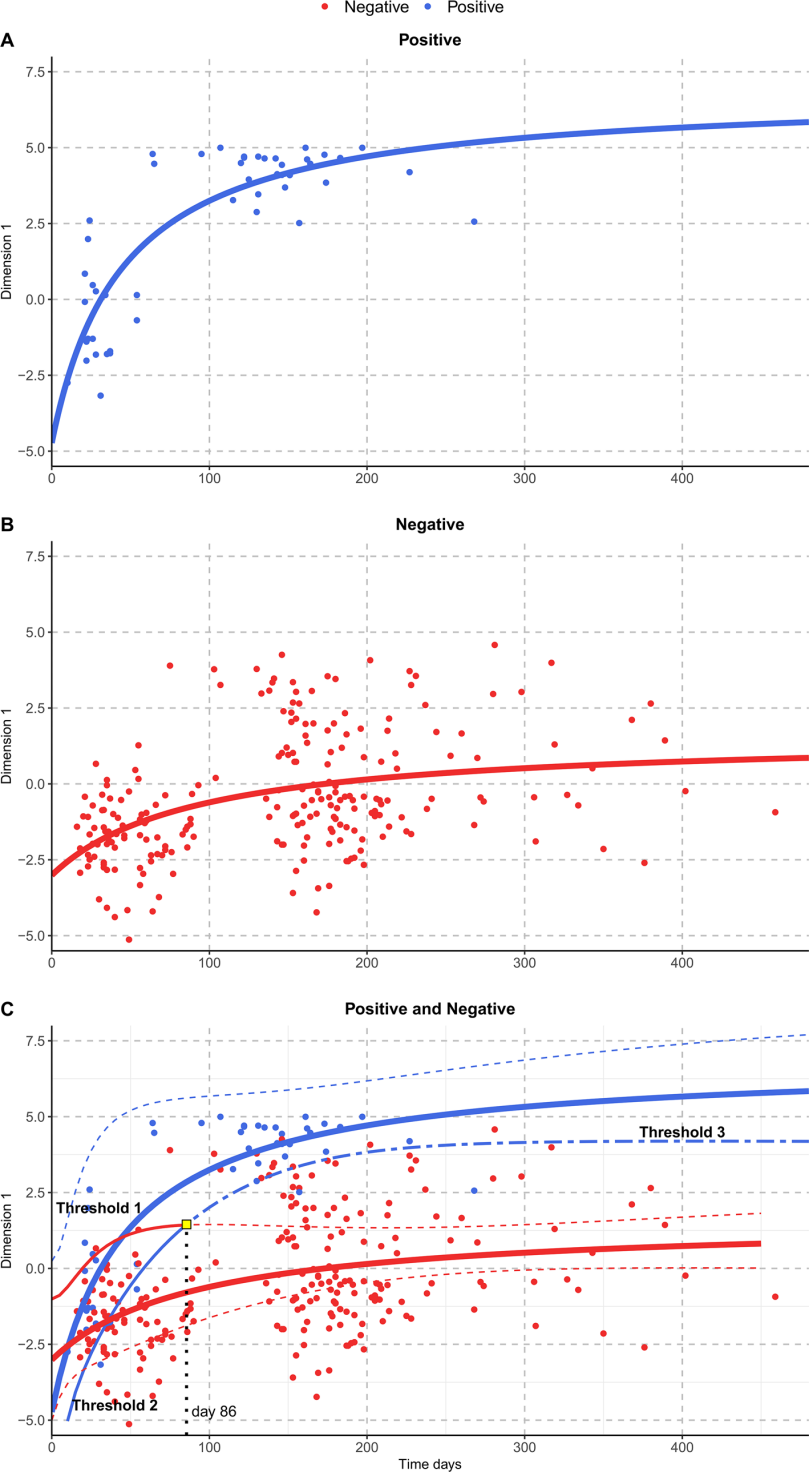
Time course of PCA Dimension 1 for individuals with Positive GOS (panel **A**) and for individuals with Negative GOS (panel **B**) in the Training sample. Dots represent the individual scores (blue dots for Positive GOS group and red dots for Negative GOS group) whereas thick continuous lines are the predictions from the Michaelis Menten model. Panel (**C**) shows the observed scores and the model fitting along with the corresponding 95% confidence intervals (dashed lines) based on a Monte Carlo approach along with the three thresholds: Threshold 1 (fine red continuous line), Threshold 2 (fine blue continuous line), Threshold 3 (blue two-dashed continuous line).

Patient scores for Dimension 1 were represented as time expressed in days after the event (Fig. 3). The time course of the Dimension 1 scores was then fitted with a Michaelis–Menten equation on the Training sample separately for individuals with positive and negative outcomes. Parameters in the positive model were identified with letter “p”; letter “n” was used for parameters in the negative model. The estimated values were: a_p_ = − 4.73 (SE = 2.47); m_p_ = 11.56 (SE = 1.49); K_p_ = 44.87 (SE = 33.73); a_n_ = − 3.01 (SE = 1.02); m_n_ = 4.61 (SE = 0.67); K_n_ = 91.99 (SE = 91.11). Although parameters a and K did not differ significantly between the two groups (*P* = 0.52 and *P* = 0.63, respectively), parameter m, indicating the maximum increment attainable for a very long time, was significantly different (*P* = 0.0001), with a greater value for the positive group. At baseline, for time equal to zero, individuals with positive and negative outcomes showed comparable values for Dimension 1. Over time, individuals with positive outcomes showed gradually increasing values. Parameter K in the positive group was on average half of that estimated for individuals with negative outcomes, which mean that their improvement was faster. Figure 3, panel C, shows the observed scores and the model fitting, along with the corresponding 95% confidence intervals (dashed lines), for both groups.

Confidence intervals were used to define cut-offs in order to identify the possible outcome of a patient, based on their clinical assessment over time. Since at baseline (a variable time in days after the event to discharge from the ICU and admission in the intensive rehabilitation unit, IRU) the values observed in the two groups mostly overlapped, a more stringent criterion was used to predict the outcome of a newly enrolled patient. Figure 3, panel C, shows that the time interval where observations are mostly superimposed and confidence intervals overlap, ranges from 0 to 86 days (which corresponds to the point where the highest limit of the negative 95% CI and the lowest limit of the positive 95% CI intersect). In this range of days, two cut-offs were used to predict the patient outcome. The first cut-off (Threshold 1, fine red continuous line) is the set of points that lie on the upper limit of the 95% CI estimated for the negative model which is used to identify subjects who will have a positive outcome if they have a higher Dimension 1 score than Threshold 1. The second cut-off (Threshold 2, fine blue continuous line) is the set of points that constitute the lower limit of the 95% CI estimated for the positive model which is used to identify subjects who will have a negative outcome if they have a lower Dimension 1 score than Threshold 2. Beyond the 86th day, the cut off becomes the lower limit of the 95% CI estimated for the positive model (Threshold 3, blue two-dashed line): individuals with Dimension 1 scores above this curve are expected to have a positive outcome. Conversely, patients are expected to have a negative outcome if their scores are below the curve. Given the chosen criteria, not all subjects can be classified if they are evaluated in the initial range (time < 86 days). Due to the superimposition of the two CIs, a certain number of patients (13/42) belong to a “shadow” area (patients with Dimension 1 scores between Threshold 1 and Threshold 2). Subsequent evaluations will predict the outcome for these subjects.

### Results of validation model

Table 3 reports the Classification Ability (number of subjects not belonging to the “shadow” area), the Accuracy, Sensitivity and Specificity of the proposed approach in the Validation sample. When patients were classified only according to the first clinical assessment performed at the IRU admission, 29 subjects were classified with an Accuracy of 89.7%, Sensitivity and Specificity of 100% and 57.1%, respectively.

**Table 3:** Accuracy and classification ability of the approach adopted.

Time points	Classification ability: number of classified individuals	Accuracy (%)	*Sensitivity (%)	*Specificity (%)	PPV (%)	NPV (%)
Validation sample (n = 42)						
T0	29/42 (69%)	89.7	100	57.1	88	100
T0 + T1	^#^40/42 (95.2%)	85	90.6	62.5	90.6	62.5
T1	^#^40/42 (95.2%)	82.5	87.5	62.5	90.3	55.6

*Computed with respect to classified subjects.

^#^The two patients who were not classified belonged to the “shadow” area for the first evaluation (time < 86 days) and presented with missing values 4 months after the event (T1).

**Figure 4:**
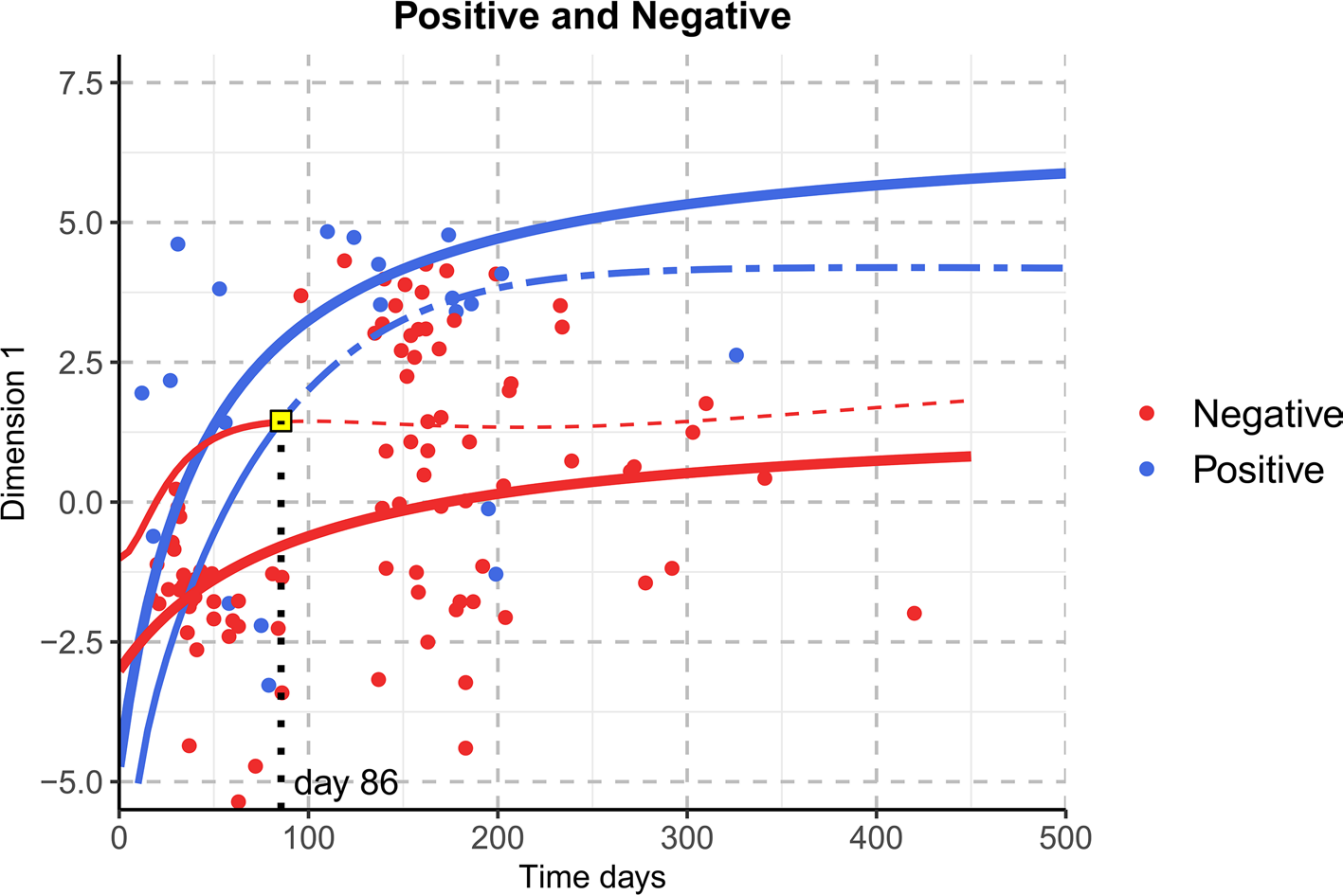
Time course of PCA Dimension 1 for individuals with Positive GOS and for individuals with Negative GOS in the Validation sample. Dots represent the individual scores (blue dots for Positive GOS group and red dots for Negative GOS group) whereas continuous lines are the predictions from the Michaelis Menten model fitted on the Training sample along with the three thresholds: Threshold 1 (fine red continuous line), Threshold 2 (fine blue continuous line), Threshold 3 (blue two-dashed continuous line).

The subsequent evaluation performed 4 months after the event provided a classification rate of 95.2% (two individuals not classified at IRU admission presented missing values at T1, and thus were unclassified) with an Accuracy, Sensitivity and Specificity of 85%, 90.6% and 62.5% respectively. If only clinical assessments at T1 were considered, the values of the indices were: 82.5%, 87.5% and 62.5%. Figure 4 shows the Dimension 1 scores regarding the Validation sample, along with the 95% CIs computed on the Training sample and used as criteria for the outcome prediction.

## Discussion

The aim of the approach proposed in this study is to identify the most determinant predictors in the clinical evolution of ABI patients and to determine their outcome trajectories. We thus combined a PCA, and a mathematical model aimed at early prediction of the GOS score, based on the estimated clinical evolution of every single patient.

The Michaelis–Menten model was used to characterize the trend of the first PCA dimension (represented by feeding modality, RLAS, ERBI-A, Tracheostomy, CRS-r and ERBI-B) in order to predict the most plausible outcome (in terms of positive or negative GOS) of ABI patients upon entry to the IRU. The proposed methodology identifies the possible evolution of the patient based on the results from the first to the subsequent clinical evaluation. Despite the greater accuracy and sensitivity (89.7% and 100%, respectively) obtained from the entrance visit to the IRU (visit at T0), for 31% of patients it was not possible to predict what their presumed outcome would be. The subsequent assessment (4-months post-event, visit at T1), however, enabled physicians to assign all patients to one of the two identified evolutions with an accuracy of 85%, a sensitivity of 90.6% and an increased specificity of 62.5% compared to 57.1% obtained with only the evaluation at T0. The only two patients for whom it was not feasible to establish the possible outcome had missing values at T1. The performance reported is in line with several previous studies using machine learning algorithms to predict outcomes in acquired brain injury^8^. In a previous study (Bruschetta et al. 2022) different ML approaches were compared with the classic Linear Model (LM) to predict the final evolution of TBI patients according to 2 and 4 GOS classes based on the clinical assessment at T0. The accuracy obtained in the case of a binary outcome was similar to that obtained with the classical LM (Bruschetta et al. 2022). The LM approach, however, underperformed in terms of sensitivity compared to our results (66.7% vs. 90.6%). The difference could be due to the additional information at T1 which determined the final outcome. Moreover, it is to be noted that our model is able to predict the evolution of a patient (in terms of outcome) whenever an observation is obtained, independently of the fact that it is a first or a second evaluation, and that the classification criterion changes after day 86 from the brain injury. This means that if a single evaluation is performed after day 86 from the event (for example for a patient with a long stay in ICU, longer than or equal to 86 days), the criterion has proven to have an accuracy of 82.5%, a sensitivity of 87.5% and a specificity of 62.5%. In this case, compared to the work of Bruschetta et al. (2022), the obtained sensitivity with our model would be superior: 87.5% versus 66.7%. As stated above, however, our model is not able to classify all patients if the first evaluation is made before day 86 from the event (prediction of the outcome was possible only for 69% of the individuals). Obviously, subsequent and additional evaluations become more informative, but again, they can be made at any point in time, and after day 86 from the event all the patients can be classified (no “shadowed” area). On the basis of the available sample of 143 individuals, the average time at T0 (first evaluation at IRU admission) was equal to 45 days (range 10–93 days), meaning that for some subjects the first evaluation is made after the intersection point and therefore these patients are classified with a sensitivity of 87.5%. The average time at T1 (second evaluation during IRU stay) was 167 days: at this point the predicted model is at 64% of its maximum, which will be reached for later dates. The average time at discharge was 207 days, about 1 month and half later than the average time at T1, a sufficient time, from a clinical point of view, to undertake a different therapeutic strategy.

The evolution over time of impairments, disabilities and recovery after ABI is characterized by a large amount of diversity^18^. Some patients show no improvement even in the long term, whereas other patients recover fully within hours or days post stroke^19^. Specific demographic (age) and clinical factors (i.e., initial severity of disability, radiological markers, comorbidities) as well as the extent of improvement observed within the first days/weeks post-injury are considered the main indicators of the outcome at 6-months^20^, although their magnitude in influencing predictive models change depending on the statistical approach^8^. In our study, we demonstrated that the indicator set of feeding modality, RLAS, ERBI-A, Tracheostomy, CRS-r and ERBI-B defined the time course of the clinical evolution in ABI patients who would be discharged with a positive or negative outcome, with the best classification of subjects and a high accuracy rate starting from the 86th day post-injury. Before the 86th day, the classification rate was low despite a very high accuracy. Although the outcome of ABI patients is heterogeneous and individual recovery patterns differ, clear mathematical regularities (i.e. logistics and sigmoidal) have been found in these nonlinear patterns of recovery, making the outcome in terms of body function and activities highly predictable^21–23^. In a recent study, van der Vliet et al.^24^, developed a longitudinal mixture model of motor recovery which describes the time course of the Fugl–Meyer motor upper extremity (FM-UE) scale after a first-ever ischemic stroke. Analyzing data from 412 first-ever ischemic stroke patients, they identified the FM-UE trajectories of 5 subgroups, organized into 3 clinically relevant clusters of poor, moderate, and good motor recovery. Their model provides a satisfactory prognosis for patients as early as 1-week poststroke, and presents some methodological differences compared to our study. First, we analyzed data from a large and heterogenous group of acquired brain injuries including vascular (50.6%), traumatic (35.9%), anoxic (9.6%) or other pathologies (3.8%). Our model then predicts the final outcome (GOS) on the basis of measurements of different clinical features: the level of consciousness (measured by CRS-r); the level of cognitive functioning (measured by the RLAS); the level of clinical complexity and disability (measured by the ERBI); the rate of paroxysmal hyperactivity of the sympathetic system (evaluated by the PSH-AM scale). The outcome is therefore estimated based on a complete clinical picture of the patient. Conversely, Vliet et al.^24^ predicted motor recovery, measured only by the FM-UE scale, and no assessment of the reliability of the model was carried out on an external cohort of subjects.

Our study has a significant limitation that needs to be addressed. Using the GOS^25^ rather than the extended version (GOSE^26^ raises concerns regarding the utility of this outcome measure for categorizing impairment. Similarly, Weir et al.^27^ demonstrated that compared to the traditional analysis, which collapses the GOS onto a binary scale (“favorable” versus “unfavorable” outcome), using an ordinal technique to evaluate the GOS significantly increases the efficiency, which is improved using the GOSE scoring. Although this methodological evaluation has only been confirmed only for the outcome assessment of TBI patients, the vast majority of clinical trials have dichotomized outcome measures of the GOS or GOSE scoring using an arbitrary cut-point^28^. Generally, any ordinal scale that is dichotomized in this way always leaves out information that might be important^29^. Hence, new, more sophisticated statistical techniques are needed to reduce the lack of the sensitivity in identifying modest but clinically significant therapy effects.

In conclusion, our combined PCA-MM approach provides an excellent prediction of patient outcome, together with the relative trend over time which demonstrates that at admission, a specific set of clinical indicators is useful to predict the outcome at discharge, however most of the dataset (no. 31) remained undefined. The prediction of the time course improves at 86 days and any new information after this time point should contribute to the understanding of functional recovery patterns in the first 3 months after injury. This study represents a significant advancement compared to our previous multicentric study using artificial intelligence algorithms^17^ to define the predictive role of traditional clinical scales. Here, the proposed mathematical modeling of ABI-related outcomes extends the static *black-box* approach (meeting the actual clinical needs) to understand how pooled well-known predictors describe the nonlinearity trajectories of clinical evolution over time in every patient. In future work, we plan to perform further external validations on other datasets in order to capture dynamic changes in prognosis during intensive care. The current model will be extended with new objective predictors, such as neuroimaging data (EEG, PET, fMRI) together with pharmacological interventions in order to explore how clinical interventions impact the outcome trajectories over time.

## Methods

### Patients

This study is a secondary analysis of data from a prospective multicenter observational study^17^, where machine learning algorithms were employed to evaluate the prognostic value of paroxysmal sympathetic hyperactivity (PSH), together with other well-known clinical features, on the outcome of ABI patients. Details of the main study are provided elsewhere^17^, however, in brief, eight neurorehabilitation units screened consecutively and admitted ABI patients with DoC from March 2018 to January 2019. Patients fulfilling the following inclusion criteria were enrolled: (1) adult age (≥ 18 years; (2) clinical diagnosis of vegetative state/unresponsive wakefulness syndrome (VS/UWS)^30^ or in a minimally conscious state (MCS, i.e. patients exhibiting minimal but definitive behavioral evidence of self-awareness or environmental awareness), and patients emerged from MCS (reliable demonstration of either interactive communication or functional object use), based on standardized clinical criteria of Coma Recovery Scale—revised (CRS-r =)[8–10]; (3) etiology: traumatic, vascular (i.e., ischemic or hemorrhagic), anoxic and other (i.e., infective or benign tumor); (4) time post-injury ≤ 3 months calculated at admission, (5) first admission in intensive rehabilitation unit (IRU). Patients with mixed etiology (e.g. anoxic and vascular), previous history of acquired brain injury, psychiatric or neurodegenerative diseases, were excluded from the study.

The present study was conducted after approval of the Institutional Ethics Committee of the coordinator center (S.Anna Institute, Protocol Register no. 244, 10/24/2017). The surrogate decision-makers of the patients enrolled in the study provided their written informed consent. The original forms were collected and stored at each participant center. All the experimental procedures were conducted according to the policies and ethical principles of the Declaration of Helsinki. Data supporting the findings of this study are included in this published article.

### Procedure and outcome measures

This was a multicenter prospective observational study. All patients underwent three specific clinical evaluations: at baseline (T0), after 4 months from the event (T1) and at discharge (T2). The examination was performed by neurologists, physiatrists or neuropsychologists with experience in disorders of consciousness who were blind to any other result. At study entry all participating centres collected: (a) demographic information, (b) etiology, (c) clinical features and presence of devices (assessment of respiratory function, tracheostomy, feeding modality, urinary catheter, pressure sores, severe spasticity, fractures, craniectomy, normal-pressure hydrocephalus). As predictive measures, we also considered the: (1) level of consciousness as measured with the Italian version of the CRS-r^31^, (2) the level of cognitive functioning as measured by the Rancho Los Amigos Scale (RLAS)^32^, (3) level of clinical complexity and disability as measured by the early rehabilitation Barthel index (ERBI)^33^. The rate of paroxysmal hyperactivity of the sympathetic system was recorded using the PSH assessment measure (PSH-AM) scale^34^. The GOS^25^ was used as the main outcome measure.

### Longitudinal Michaelis–Menten model

The GOS score, originally coded into 5 levels (death = 1; vegetative = 2; severe disability = 3; moderate disability = 4; good recovery = 5) was categorized into a binary variable: “positive outcome” (GOS ≥ 4) and “negative outcome” (GOS < 4), following previous criteria^35,36^.

For the analysis purpose, the entire sample was split into a Training (70% of cases) and a Validation (30% of cases) sample, by using the data-splitting method balanced according to the GOS variable. The Training sample was used as the fitting population for model development. The performance of the model was tested in the Validation sample.

The set-up procedure employed, as a first step, a Principal Component Analysis (PCA) in the Training sample with the aim of reducing the dimensionality of the dataset. A preliminary univariable analysis was performed to identify predictors significantly associated with GOS outcome at each evaluation time point. Given the presence of both continuous and categorical variables, a Factorial Analysis of Mixed Data (FAMD) approach was used. Continuous variables were scaled to unit variance and the categorical variables were transformed into a set of disjunctive variables (0 in absence of the modality; 1 in presence of the modality) and then scaled. The first two principal components, explaining most of the dataset variability, were then interpreted based on the scores that each predicting factor exhibited on each component. The variables entered the PCA with all the available clinical evaluations (baseline, T0; after 4 months from the event, T1; at discharge, T2). This allowed us to explore the evolution over time of the PCA dimensions. The trend of the dimensions was evaluated according to the positive/negative outcome. Subjects were represented on a two-dimensional space according to their values on the first 2 PCA components. The first component was then evaluated over time and fitted with a Michaelis–Menten function, which exhibits a saturated trend with increasing dependent variable $x$:

$$f\left(x\right)=a+\frac{m*x}{K+x}$$

where parameter $a$ is the value of the dependent variable (Dimension 1) at time 0 ($x=0$); $m$ is the increment that the dependent variable takes to achieve its maximum value ($a+m$) as ***x*** goes to infinity; ***K***, the Michaelis constant, is the value of variable ***x*** at which the function reaches the value $a+m/2$. In the function $f\left(x\right)$ the independent variable $x$ represents the ***time.***

### Model fitting

The parameter vector ($a,m,K$) was estimated by minimizing the sum of squared residuals by using the adaptive non-linear least squares algorithm. The model was adapted separately to observations from individuals with positive and negative outcome with the aim of assessing two possible different evolutions for these two subgroups of patients.

Parameter estimates were identified for each subgroup (positive/negative outcome), average trends and the corresponding 95% confidence intervals were then obtained. Confidence intervals for the fitted values were computed by means of a Monte Carlo approach by drawing 10,000 samples from multinormal distributions on the model parameters.

### Model-validation

The coordinates on the PCA components (built on the Training dataset) were predicted on the Validation sample. Confidence intervals for the fitted values of the two Michalis–Mentel models (positive/negative GOS outcome) on the Training dataset were used to identify thresholds for predicting the evolution over time of the individuals in the Validation dataset. Thresholds were established at different time points and their predictive ability in determining a positive/negative GOS outcome was assessed by computing accuracy, sensitivity and specificity.

### Statistical analysis

Means and standard deviations and counts and percentages were used to summarize continuous and categorical variables, respectively. Chi-squared test, or Fisher exact test when appropriate, was used to study the possible association between factorial variables and the positive/negative GOS outcome at each time point. For continuous variables a two-independent samples t-test was used to assess possible differences between the two groups. All statistical analyses were performed with R package version 4.1.3.

### Study registration

This study was approved by the Ethical standards Committee of the coordinator center as a multicenter observational study (Protocol Register n. 244, 10/24/2017).

## Supplementary Information

Supplementary Table 1.

Supplementary Legends.

Supplementary Table 2.

## Data Availability

All data generated or analysed during this study are included in this published article [Supplementary Table 2.xls].

## Abbreviations

GOS: Glasgow outcome scale
CRS-r: Coma recovery scale-revised
ERBI: Early rehabilitation Barthel index
ICU: Intensive care unit
IRU: Intensive rehabilitation unit
RLAS: Rancho Los Amigos scale
MCS: Minimally conscious state
ML: Machine learning
MM: Michaelis–Menten
PSH-AM: Paroxysmal sympathetic hyperactivity-assessment measure
ABI: Acquired brain injury
VS/UWS: Vegetative state/unresponsive wakefulness syndrome
PCA: Principal component analysis
FAMD: Factorial analysis of mixed data
