# Supplementary material for: Modeling outcome trajectories in patients with acquired brain injury using a non-linear dynamic evolution approach
Source: Sci Rep. 2023 Apr 18;13:6295. doi: 10.1038/s41598-023-33560-x (PMC10113248; doi:10.1038/s41598-023-33560-x)
Supplement: Supplementary file 1 — Supplementary Table 1. [file 41598_2023_33560_MOESM1_ESM.docx]

| **Clinical Variables** | **T0 (admission)** | **T1 (4 months)** | **T2 (discharge)** |
| --- | --- | --- | --- |
| Diagnosis (n°, %)   - VS/UWS - MCS - Emerged from MCS - Died | 61 (39.2%)  55 (35.2%)  40 (25.6%)  0 (0%) | 31 (21.6%)  32 (22.2%)  79 (54.8%)  2 (1.4%) | 19 (13.2%)  33 (22.9%)  88 (61.1%)  4 (2.8%) |
| CRS-r | 9 [1-23] | 17 [1-23] | 21 [3-23] |
| RLAS | 3 [1-7] | 4 [2-8] | 5 [1-8] |
| ERBI | -175 [-50 /-325] | -175 [0 /-325] | -100 [0 /-175] |
| PSH-AM score | 5 [0-23] | 3 [0-21] | 1 [0-18] |
| PSH signs (n°, %)   - None [0-7] - Possible [8-16] - Probable [>17] | 107 (68.7%)  31 (19.8%)  18 (11.5%) | 118 (83.4%)  15 (9.6%)  11 (7%) | 137 (95.6%)  4 (2.5%)  3 (1.9%) |
| Feeding (n°, %)   - PF - NGT - PEG - Oral | 3 (1.9%)  63 (40.4%)  79 (50.6%)  11 (7.1%) | 1 (0.6%)  11 (7.6%)  74 (51.4%)  58 (40.3%) | 2 (1.3%)  3 (2.1%)  69 (47.9%)  70 (48.6%) |
| Breathing condition (n°, %)   - Assisted - Autonomous | 5 (3%)  151 (97%) | 1 (0.6%)  143 (99.4%) | 1 (0.6%)  143 (99.4%) |
| Tracheostomy (Yes, %) | 137 (88%) | 80 (55.5%) | 52 (36.1%) |
| Spasticity (Yes, %) | 34 (21.8%) | 48 (33.3%) | 46 (31.9%) |
| Urinary catheter (Yes, %) | 150 (96.1%) | 64 (44.4%) | 48 (33.3%) |
| Bedsores (Yes, %) | 58 (37.2%) | 41 (28.4%) | 24 (16.6%) |
| Craniectomy (Yes, %) | 34 (21.8%) | 25 (17.3%) | 14 (9.7%) |
| Hydrocephalus (Yes, %) | 15 (9.6%) | 26 (18%) | 21 (14.5%) |

**Supplementary Table 1. Clinical evolution during IRU period**

*ICU: intensive care unit; IRU: intensive rehabilitation unit; LOS: length of stay; VS/UWS: vegetative state/unresponsive wakefulness syndrome; MCS: Minimally Conscious State; IRU: Intensive Rehabilitation Unit; PF: parenteral feeding; PEG: percutaneous endoscopic gastrostomy; NGT: nasogastric tubes. CRS-r: Coma Recovery Scale-Revised (CRS-R), RLAS: Racho Los Amigos Scale; ERBI: Early Rehabilitation Barthel Index (ERBI); PSH-AM: Paroxysmal Sympathetic Hyperactivity-Assessment Measure.*
