# Supplementary material for: Modeling outcome trajectories in patients with acquired brain injury using a non-linear dynamic evolution approach
Source: Sci Rep. 2023 Apr 18;13:6295. doi: 10.1038/s41598-023-33560-x (PMC10113248; doi:10.1038/s41598-023-33560-x)
Supplement: Supplementary file 2 — Supplementary Legends. [file 41598_2023_33560_MOESM2_ESM.docx]

**Legend Supplementary Materials Table S2**

**Modeling outcome trajectories in patients with acquired brain injury using a non-linear dynamic evolution approach.**

Simona Panunzi^1*^, Lucia Francesca Lucca^2*^, Antonio De Tanti^3^, Francesca Cava^4^, Annamaria Romoli^5^, Rita Formisano^6^, Federico Scarponi^7^, Anna Estraneo^5^, Diana Frattini^8^, Paolo Tonin^2^, Ilaria Piergentilli^1^, Giovanni Pioggia^9^, Andrea De Gaetano^1,9,10^, Antonio Cerasa^2,9,11§^

Legend Table S2: ICU: Intensive Care Unit; CRS-r: Coma Recovery Scale-Revised; RLAS: Rancho Los Amigos Scale; ERBI: Early Rehabilitation Barthel Index; PSH-AM: Paroxysmal Sympathetic Hyperactivity-Assessment Measure; CFS: clinical feature scale; DLT: diagnosis likelihood tool. TBI: Traumatic Brain Injury; MCS: Minimally Conscious State. VS/UWS: Vegetative State/unresponsive wakefulness syndrome; PEG: percutaneous endoscopic gastrostomy.; NG tube: nasogastric tube.
